# The Neuroprotective Effects of Long-Term Repetitive Transcranial Magnetic Stimulation on the Cortical Spreading Depression-induced Damages in Rat’s Brain

**DOI:** 10.29252/NIRP.BCN.9.2.87

**Published:** 2018

**Authors:** Babak Khodaie, Valiallah Saba

**Affiliations:** 1. Department of Radiology, Faculty of Paramedicine, AJA University of Medical Sciences, Tehran, Iran.; 2. Shefa Neuroscience Research Center, Khatam-Alanbia Hospital, Tehran, Iran.

**Keywords:** Cortical Spreading Depression (CSD), Repetitive Transcranial Magnetic Stimulation (rTMS), Apoptosis, Cortex, Hippocampus

## Abstract

**Introduction:**

Cortical Spreading Depression (CSD) is a propagating wave of neural and glial cell depolarization with important role in several clinical disorders. Repetitive Transcranial Magnetic Stimulation (rTMS) is a potential tool with preventive treatment effects in psychiatric and neuronal disorders. In this paper, we study the effects of rTMS on CSD by using behavioral and histological approaches in hippocampus and cortical regions.

**Methods:**

Twenty-four rats were divided into four groups. A group of control rats were kept in their home cage during the experiment. The CSD group received four CSD inductions during 4 weeks with 1 week intervals. The CSD-rTMS group were treated with rTMS stimulation (figure-eight coils, 20 Hz, 10 min/d) for 4 weeks. The fourth group, i.e. rTMS group received rTMS stimulation similar to the CSD-rTMS group without CSD induction.

**Results:**

Long-term rTMS application in treated groups significantly reduced production of dark neurons, increased the mean volume of normal neurons, and decreased the number of apoptotic neurons in cortical regions compared to the control group. The protective effects of long-term treatment by rTMS in the hippocampal regions were also studied. It was effective in some regions; however, rTMS effects on hippocampal regions were lower than cortical ones.

**Conclusion:**

Based on the study results, rTMS has significant preventive and protective effects in CSD-induced damages in cortical and hippocampal regions of the rat’s brain.

## 1. Introduction

Cortical Spreading Depression (CSD) is a propagating wave of neural and glial cell depolarization that spreads out over the cerebral cortex at a velocity of 3–5 mm/min (Leao, 1944). CSD spreads centrifugally from the site of initiation as a radial wave across the neuronal tissue (Haghir, Kovac, Speckmann, Zilles, & Gorji, 2009). CSD-like waves were detected during appearance of visual aura in migraine attacks (Hadjikhani et al., 2001) and also involved in progressive neural death in stroke and other neurological disorders, like acute brain trauma (Strong et al., 2002; Fordsmann et al., 2013). Migraine is a disabling neurological disorder characterized by unilateral head pain. It affects 16% of worldwide population, and about one third of migraine cases are suffering from a neurological symptoms associated with a transient cortical malfunction, known as aura (Noseda & Burstein; 2013). The relationship of such a cortical distribution and cortical spreading depression has been previously described (Gorji, 2001).

In addition, effects of CSD phenomenon in cerebrovascular diseases, head injury, and transient global amnesia have been reported (Gorji, 2001). Occurrence of CSD is observed in various cerebrovascular diseases (including intracranial hemorrhage, stroke, and subarachnoid hemorrhage). Moreover, clinical and experimental studies reported that CSD may have a role in epilepsy and transient global amnesia as well as some spinal cord disorders (Ghaemi et al., 2014). Dreier et al. (2007) have shown that in contrast to CSD under physiological conditions, CSD waves are reported to be related to neuronal death under pathological conditions. Neuronal death due to repeated CSD in intact juvenile brains may be related to the pathogenesis of some neurological disorders in infants and children (Somjen, 2006; Lotfinia et al., 2014). Furthermore, changes in the energy demand during CSD will lead to an imbalance between supply and demand, resulting in neuronal injury in juvenile rats and not adults (Sadeghian et al., 2012). It has been previously postulated by numerous studies that dark neurons could represent a typical morphological change of injured neurons caused by various injuries (Ooigawa et al., 2006; Jafarian et al., 2010). Significant increase of the density of necrotic cell was previously reported in hippocampal and cortical region of rats after repetitive CSD induction (Jafarian et al., 2010).

Function of hippocampus could be impaired by neuronal damage. As previous studies confirmed that hippocampus is concerned with imprinting and early recall of memories (Turner, 1969), studying of memory performance could be important in understanding of hippocampus functional activity. CSD is triggered by elevation of extracellular potassium and or glutamate level (Grafstein, 1956), resulting in large field potential shift and increasing ion distributions (Somjen, 2001) along with cellular excitation. Propagation of CSD is accomplished by the release and diffusion of several chemical mediators, including excitatory amino acids, calcitonin gene-related peptide, neurokinin, serotonin, and brain-derived neurotrophic factor into the inter-stitial space. This could change the receptors affinities and subsequently alter the neuronal network activities (Gorji, 2001). Up-regulation of the glutamate binding sites was observed in rat neocortex, hippocampus, and striatum, enhancing neuronal excitability and reducing the threshold for CSD initiation (Haghir et al., 2009; Martens-Mantai, Speckmann, & Gorji, 2014). Studies indicate that intracortical inhibition can be dramatically impaired by repetitive CSD induction, caused by reduction in GABAergic inhibition and changes in excitatory field potential (Krüger, Heinemann, & Luhmann, 1999).

Transcranial Magnetic Stimulation (TMS) was first used by Barker in 1985. In some neurological disorders, e.g. Parkinson disease, epilepsy, migraine, and chronic pain, rTMS has showed a potential therapeutic effect (You, Tzschentke, Brodin, & Wise, 1998; Fregni & Pascual-Leone 2007). However, the effects of rTMS depend on the frequency of radiation. High frequency (i.e. 20 Hz) stimulation causes an enhancement of brain excitability while low frequency (i.e. 1 Hz) could lead to reduction of brain excitability (Gangitano et al., 2002; Romero, Anschel, Sparing, Gangitano, & Pascual Leone 2002; Fregni et al., 2005; Fadakar, Saba, & Farzampour 2012). Although, some other studies have suggested that both high and low frequencies behave in the same way and increase brain excitability (Fregni et al., 2005).

TMS-induced electrical current passing through subjects’ scalps and skulls without attenuation (Pascual-Leone, 1999), provides sustained interruption in neuronal activity, changes cortical metabolisms and cerebral blood flow (George et al., 1997). TMS could trigger a group of neural range in the stimulated area of brain, and this can be followed by a mixture of both inhibitory and excitatory effects (Siebner & Rothwell, 2003). So, rTMS is an efficient tool for reducing ischemic neuronal damages and may provide neuroprotective effects (Fujiki, Kobayashi, Abe, & Kamida. 2003). Results obtained from previous studies indicate that rTMS increases the GABAergic inhibition (Pascual-Leone, Valls-Solé, Wassermann, & Hallett, 1994; Hallett, 2000). Appropriate intensity and frequency of rTMS application could promote intracortical inhibition (Fregni & Pascual-Leone 2007). The effects of rTMS in different neurological and psychiatric disorders have been studied; however, there are few studies indicating the efficacy of rTMS in behavioral, structural, and functional damages of the brain caused by CSD. In the current work, the protective effects of rTMS in reducing neuronal injuries in a rat model of CSD have been studied. The study procedure and tools are described in the next sections.

## 2. Methods

### 2.1. Animals

Twenty-four juvenile male Wistar rats (25–35 days old; 45–90 g) were used in the current study. Animals were maintained in a reversed 12-h light/dark cycle and housed at the normal temperature of 22°C±2°C. Food and drinking water was available ad libitum. Animals were divided into four groups, including control, CSD, rTMS-CSD and control rTMS groups for memory and histological evaluation. Control rats stayed in their home cage during experiment. The rats in CSD group received four consecutive induction of CSD. We applied rTMS stimulation in both rTMS-CSD and rTMS groups for 4 weeks. In addition, rTMS-CSD group received four consecutive CSD during treatment period.

### 2.2. Surgery

Rats were anesthetized with ketamine (150 mg/kg; Sigma, USA). The surgical method has been explained in detail previously (Khodaie et al., 2015). Similar approach was used by employing stereotaxic instrument (Stoelting Instruments, USA) at the same coordinates (Paxinos & Watson, 2006), including “anterior-posterior=+1.8 mm anterior to the bregma; medial-lateral=−3.1 mm lateral to the sagittal suture; dorsal-ventral: 0.7 mm” down from the skull surface to implant a guide cannula. A stylet was used to maintain patency of guide cannula for future injection. Silver electrodes were also placed (without injuring the dura mater) over the somatosensory cortex of all studied animals with a reference electrode over the nasal bulb. Dental acrylic cement was used to fix the electrodes and cannula at the implanted regions. The scalp was sutured and animals were returned to their home cages. All animal were kept in well-ventilated boxes for a week to recover from surgery.

### 2.3. Repetitive transcranial magnetic stimulation

A commercially available stimulator (TAMAS, South Korea) which was equipped by a -eight coil, including an external loop diameter of 7 cm was used for rTMS. A biphasic waveform with a pulse width of 350 μs was used for experiment. The coil temperature and the stimulation intensity were monitored from a digital display. The coil was placed parallel to the skull of the rat and the distance between the rat’s head and the coil was maintained at 1 cm. During stimulation, all animals were examined while awake and restricted to confirm that they remained calm and comfortable during the stimulation periods. The rTMS was administrated at a rate of 20 Hz for 2 s with 28 s inter-train intervals (2.1 T, 70% of the maximal output of the stimulator), repeated 10 times. The treatments were performed in 5-day series separated by 2-day intervals for 4 weeks (Fleischmann, Hirschmann, Dolberg, Dannon, & Grunhaus, 1999; Li, Yang, Ye, Yang, & Wang, 2007). All rTMSs were administered between 9:00 and 11:00 in the morning (Müller, 2000).

### 2.4. CSD induction

Rats were anesthetized using pentobarbital (Sigma; 60 mg/kg, IP), then the stylet was removed from the guide cannula and a proper injection needle was inserted. A 10 μL Hamilton syringe was attached to a polyethylene tube (Harvard Apparatus, Inc.). About 3 M KCl solution was injected in a total volume of 10 μL within a minute (n=12). The injection needle was kept in the guide cannula for an additional minute following the injection to aid complete diffusion of the KCl. In sham injected animals (n=12), 10 μL of Ringer solution was used to inject according to the same procedure. Four consecutive induction of CSD was carried out (with an interval of 1 week). Anesthesia was maintained for 60 min after injection of KCl or Ringer solution (Ghaemi et al., 2014).

### 2.5. Electrocortocencephalogram (ECoG) recordings

ECoG was recorded by silver electrodes connected to an amplifier (EXT-02 F, NPI, and Germany). Signals were digitalized by Digidata 1440A Digitizer; Axon CNS instrument. ECoG waves were analyzed by Axo-scope 10.2 software (Axon CNS Instrument, Inc. USA) (Khodaie et al; 2015).

### 2.6. Passive avoidance task

Seven, six, and one day before perfusion, the rats in all groups were subjected to a memory retention deficits test using a passive avoidance apparatus. The apparatus had two compartments (25×15×15 cm high), dark/light shuttle boxes, with a guillotine door separating two compartments. The dark compartment was equipped with a stainless steel shock grid floor. Passive avoidance test was examined during three days. Adaptation and learning was performed on the first day of testing. For adaptation, each animal was placed in the light chamber and left there for 5 minutes to be habituated. After a 10-s habituation period, the guillotine door was removed, and the first latency period of the animals to enter the dark chamber was recorded. Animals with an initial latency period of 60 s or more were excluded from further experiments. For learning steps, after the animals had entered the dark chamber, the guillotine door was closed immediately and an electric foot shock (1 mA, 50 Hz) was delivered to the floor grid for 1 s. Twenty seconds later, the rat was removed. This training phase was continued until the rat stayed in the light compartment for 300 s. The first retention trial was carried out 30 minute after the training trial and was performed in the same way as in the training trial, but the foot shock was not performed and the step-through latency was recorded for up to 300 s. The second retention trial was performed one day after the training trial. The third retention trial was performed one week after the training trial. Shorter latencies indicate poorer retention (Miyamoto, Shintani, Nagaoka, & Nagawa, 1985).

### 2.7. Histopathology assessment

**Figure 1.**
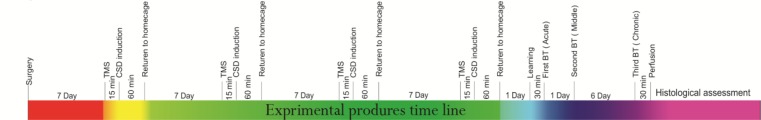
Time line of experimental procedure; Providing a schematic line to describe different stages of experiment Four consecutive CSD induction followed by recovery in home cage and at the end of CSD induction, the rat were perfused and histological study were done

Four weeks following repetitive application of KCl (CSD and rTMS-CSD groups) or Ringer solution (control and rTMS groups) with an interval of one week, and completion of passive avoidance test, the rats were perfused and brain slices were prepared as described below. All rats were given a deep anesthesia with chloral hydrate (350 mg/kg; Sigma-Aldrich) and perfused transcardially with 150 mL of saline followed by 600 mL of 4% paraformaldehyde (PFA) solution. The brains were removed and kept in 4% PFA for about 1 week. Coronal serial 8-μm sections were obtained and stained by toluidine blue. Hippocampal areas, including CA1, CA3, Dentate Gyrus (DG), and cortical regions, including somatosensory (S.s. cortex) and entorhinal (Ento. cortex) were examined using a light microscope (BX51, Olympus, Japan) connected to a digital camera. Digital photographs were captured using a 40× objective lens (Olympus, Japan). The magnification was calculated using an objective micrometer (Khodaie et al., 2015). Figure 1 displays the timing of different experimental procedures.

### 2.8. Neuronal apoptosis assay

To detect DNA fragmentation, Terminal Deoxynucleotidyl Transferase (TdT) - Mediated dUTP Nick-End Labelling (TUNEL) was used for staining DNA fragmentation after apoptotic cell death employing an in situ Cell Death Detection Kit (Roche, Mannheim, Germany), as explained previously (Otsuki, Li, & Shibata, 2003). In brief, three 8-μm thickness tissue sections were selected from each block and then dewaxed and rehydrated by heating at 60°C continued by washing in xylene, and rehydration by diluted alcohol. Followed by washing with 10 mM Tris–HCl (pH=7.6), the tissues were incubated in methanol containing 0.3% H_2_O_2_ for 10 min to restrict and prohibit endogenous peroxidase action. The sections were then covered by proteinase K (Roche, 20 μg/mL in Tris buffer) at 37°C for 30 min. TUNEL reaction mixture (450 μL of label solution and 50 μL of enzyme solution) were used for incubation of sections at 37°C for 60 min and in POD solution for 30 min. In order to develop color reaction, we used 3-3′-diaminobenzidine (DAB, Roche; 0.5 μL DAB and 1.5 μL peroxide buffer) for 5–10 min, then hematoxylin was employed for counterstaining. The percentage of TUNEL-positive cells was analyzed by counting 500 cells in each specimen (Sadeghian et al., 2012).

### 2.9. Stereological methods and physical dissector

The volume-weighted (a mathematical method used to increase the accuracy of the measurements) mean volume of normal cells was evaluated directly by point-sampled intercept on 10 uniform systematic randomly pictured microscopic fields (Staubli, Rogers, Lynch, 1994, Haghir et al., 2009). The volume-weighted mean volume of neuronal cells was calculated in CA1, CA3, dentate gyrus, somatosensory, and entorhinal cortical regions. The mean volume-weighted of neurons was calculated by the point sampled intercept technique (Gundersen et al., 1988). A test system involving parallel lines related to test points was superimposed on each microscopic field. The path of the lines on the sample was evaluated by lottery. For every point inside the unbiased determined border frame, which smashes a nucleus, the nuclear intercept by the point was considered.

A network of test points on lines was superimposed arbitrarily on to the traced nuclear profiles in each particular tissue field. Nuclei of neurons were clarified and two isotropic lines from randomly designated guidelines were centered on this neuron and superimposed. We marked the intersection of every line with the outer surface of the neuronal soma. Aforementioned lines formed point-sampled intercepts whose lengths were calculated, cubed, then their mean was multiplied by π/3, and finally the averaged volume-weighted mean neuronal volume was estimated. For quantitative calculation of dark neurons, the physical dissector technique was used. We selected ten pairs of tissue sections, with 5 mm distance, for each brain. We considered the first section as a reference and the second one was used for comparison. On each selected and pictured pair of sections, at least 10 microscopic fields were considered by uniform systematic random sampling in each area of interest (Gundersen et al., 1988).

### 2.10. Statistical analysis

Data were presented as mean±SEM. Statistical analysis was done by using 1-way analysis of variance (ANOVA) which then continued by Tukey post hoc test. The criterion for statistical significance was P<0.05. We also used the Pearson test to analyze the correlation between the number of necrotic cells and the volume of neurons.

## 3. Results

### 3.1. Electrophysiological analyses

**Figure 2.**
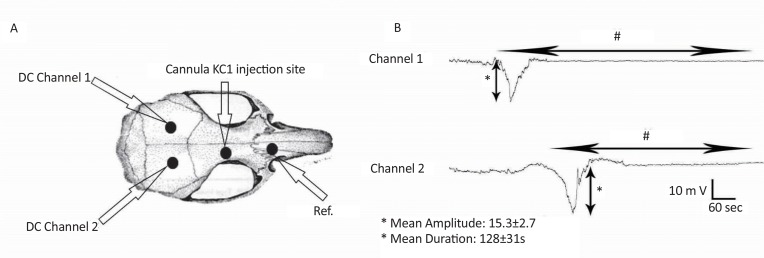
Recordings of cortical spreading depression following KCl injection (3 mol/L) in rat brain A: As indicated, two silver recording electrodes (2–3 mm apart) were implanted above the cortex and a guide cannula was used for injection and then fixed. Negative fluctuation was recorded from the somatosensory cortex of rats. B: CSD wave after KCl induction indicates negative DC deflection. In addition using two channel of recording resulted in calculation of CSD velocity

Injection of KCl into the brain resulted in negative DC fluctuations, which were continued by positive waves in CSD-induced animals. Figure 2 shows the mean wave duration (128±31 s) as well as amplitude (15.3±2.7) of the neocortical SD-like waves, which showed significant changes compared to normal ECOG wave in base line. However, no significant change in the amplitude, duration, and the speed of propagation of SD-like waves during four weeks was observed compared to CSD features in the first week.

### 3.2. Passive avoidance task

**Figure 3.**
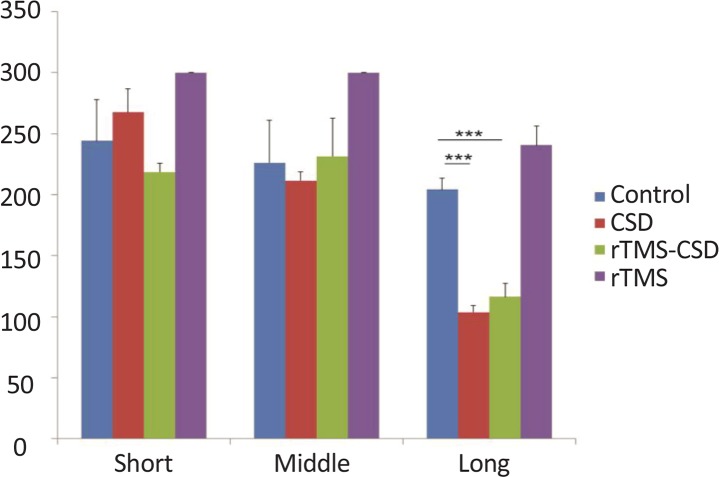
Passive avoidance test results presented during one week after CSD induction and rTMS stimulation Significant decrease in mean latency time was observed in CSD and treated animal in long term avoidance test. There is no significant change in short- and middle-term test compared to the control animals. Values are presented as Mean±SEM. *P<0.05; ** P<0.01; *** P<0.001

According to Figure 3, shuttle-box avoidance latency showed no significant difference in short (30 min after training) and middle term (24 hours after training) tests between different groups. However, significant reduction in both CSD and treated groups was observed in long-term (7 day after training) test compared to the control animals (P<0.001) (Figure 3). Table 1 presents the mean time of latency in shuttle test.

**Table 1.** Mean time (s) of latency in shuttle test in different groups

**Groups**	**Mean±SEM**		

	**Short Term**	**Middle Term**	**Long Term**
Control	244±33.93	226±34.83	204.2±9.39
CSD	267±19.3	211.52±7.46	103.75±5.2
rTMS-CSD	218.5±7.39	231.5±30.9	116.2±10.8
rTMS	300±0	300±0	240.5±15.75

### 3.3. The mean number of necrotic cells

**Figure 4.**
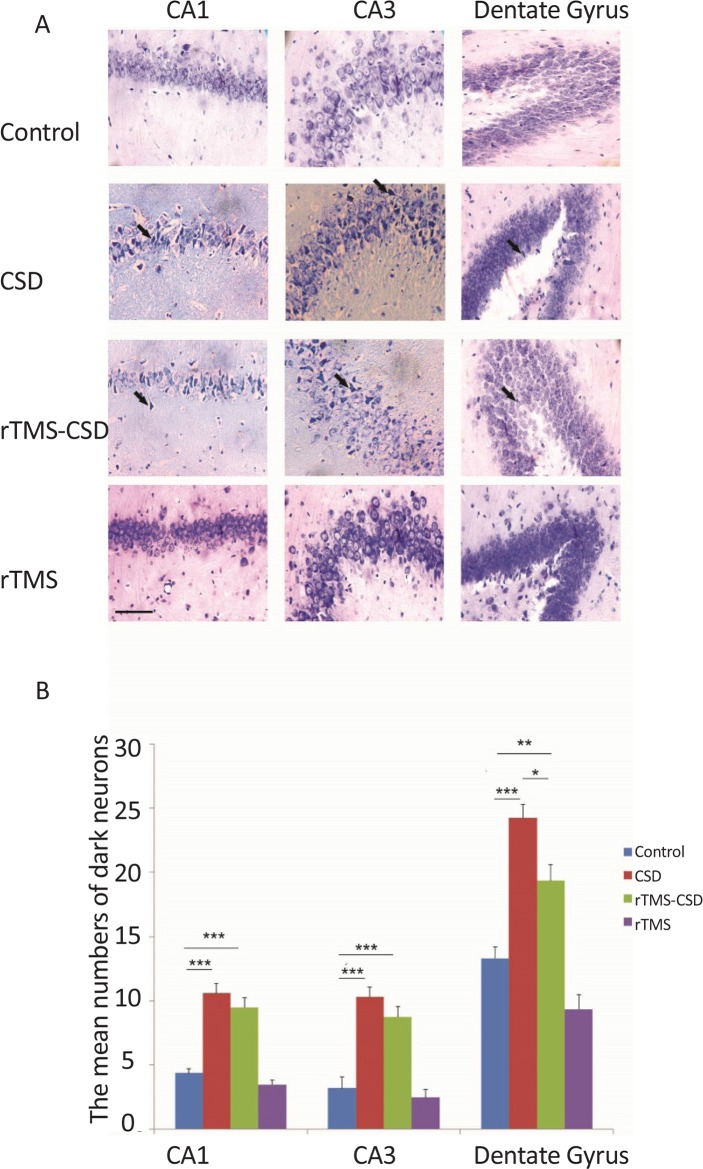
The effect of CSD induction and rTMS stimulation on the number of necrotic cells in CA1, CA3, as well as dentate gyrus region A: Light-microscopic appearance (40× magnification) of coronal brain section in the control, CSD, rTMS treated animals as well as rTMS control in all studied areas; B: The bar graph indicates that the mean dark neurons number in CSD and rTMS groups have significantly increased in all experimental areas compared to the control animals. However, significant reduction in the mean number of necrotic cells was observed in hippocampal areas of rTMS-CSD group compared to CSD animals. Scale bar: 50 μm .Values are presented as Mean±SEM. * P<0.05; ** P<0.01; *** P<0.001

Necrotic cells were diagnosed by the “neuronal shrinkage, cytoplasmic eosinophilia, nuclear pyknosis, and surrounding spongiosis” (Khodaie et al., 2015). Figure 4A shows the production of necrotic cells in studied regions of the hippocampus; CA1 and CA3 as well as dentate gyrus. Furthermore, the same study was done in the somatosensory and entorhinal cortical areas (Figure 4A). Histological studies indicated a significant increase in the mean number of necrotic cells in the CA1 and CA3, dentate gyrus region of the hippocampus in CSD group compared to the control rats (P<0.00) (Figure 4B). Moreover, the mean number of necrotic cells in dentate gyrus region of the hippocampus was significantly lower in rTMS-CSD rats compared to the CSD group (P<0.05) (Figure 4B). Significant higher mean number of necrotic cells was also observed in CA1 and CA3, and dentate gyrus areas of the hippocampus in rTMS-CSD group compared to the control animals (P<0.001, P<0.001, P<0.01, respectively) (Figure 4B). Table 2 presents the mean number of dark neurons.

**Table 2.** The mean number of necrotic cells in different brain regions of experimental groups

**Groups**	**Mean±SEM**				
	
**Regions**	**CA1**	**CA3**	**Dentate Gyrus**	**S.s Cortex**	**Ento Cortex**
Control	4.36±0.35	3.2±0.84	13.29±0.87	2.25±0.4	3.17±0.27
CSD	10.59±0.74	10.3±0.75	24.22±1.09	5.51±0.58	6.77±0.72
rTMS-CSD	9.4±0.77	8.7±0.81	19.33±1.25	3.7±0.17	4.81±0.3
rTMS	3.47±0.34	2.4±0.64	9.34±1.13	2.08±0.2	3.36±0.33

**Figure 5.**
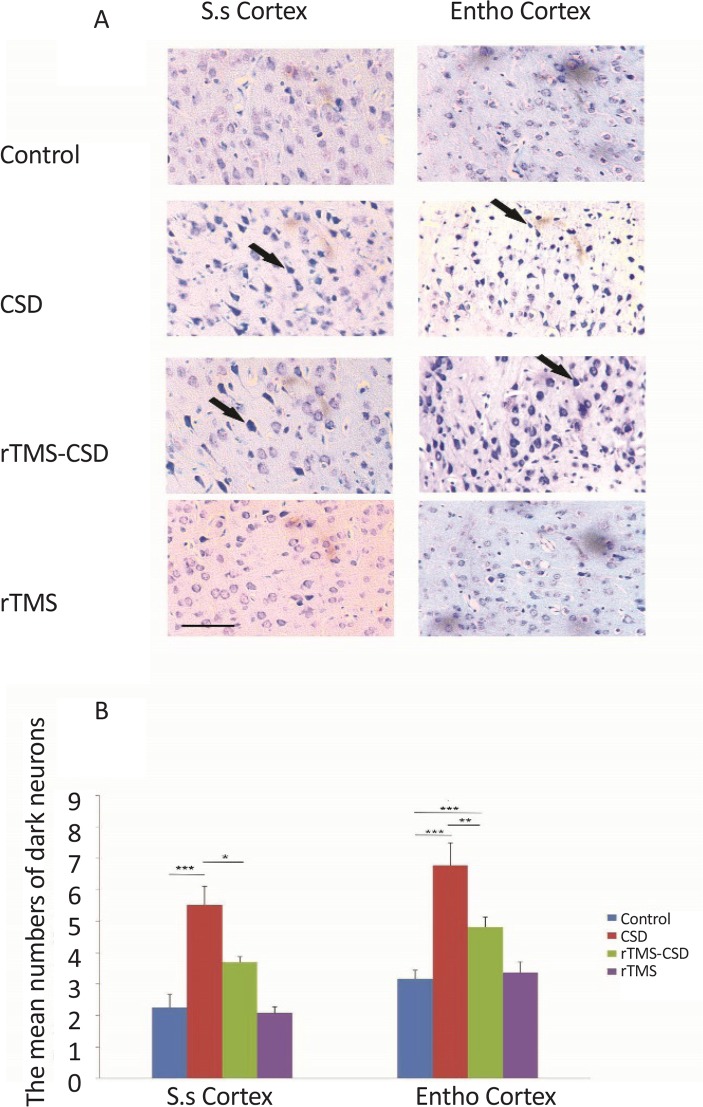
The effect of CSD induction and rTMS stimulation on the number of necrotic cells in somatosensory and entorhinal cortical region A: Light-microscopic appearance (40× magnification) of coronal brain section in the control, CSD, rTMS treated animals as well as rTMS control in all studied areas; B: The bar graph indicates that the mean number of dark neurons in the CSD group significantly has increased in both studied areas compared to the control animals. However, significant reduction in the mean number of necrotic cells was observed in both cortical areas of rTMS-CSD group compared to CSD animals. Scale bar: 50 μm. Values are presented as Mean±SEM. * P<0.05; ** P<0.01; *** P<0.001

There was a significant increase in the mean number of necrotic cells in both cortical somatosensory and entorhinal cortex regions of the CSD group compared to the control animals (P<0.001) (Figure 5B). Furthermore, studies showed a significant increase in the mean number of necrotic cells in entorhinal cortical region of treated animals in rTMS-CSD groups compared to the control animals (P<0.01). However, treated animals in rTMS-CSD groups in both studied areas of cortex also revealed a significant reduction in the mean number of necrotic cells compared to the CSD group (P<0.05, P<0.01) (Figure 5B).

### 3.4. Neuronal apoptosis

**Figure 6.**
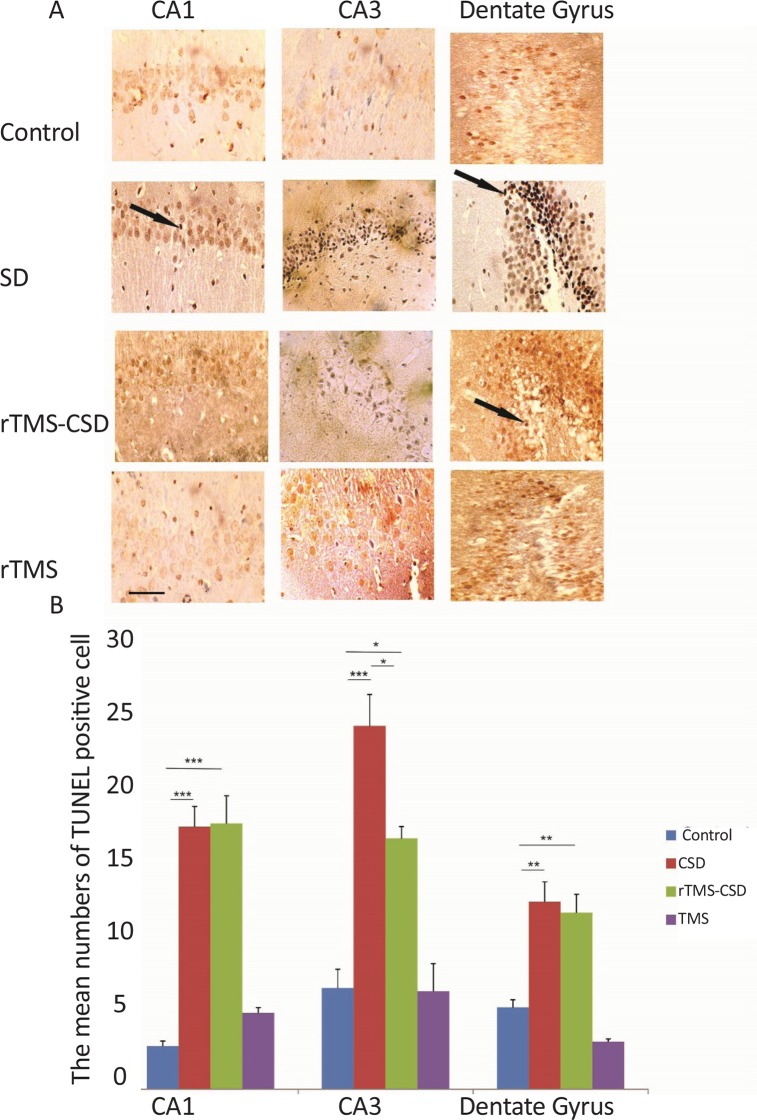
Positive TUNEL-reacted indicative of apoptotic cell death in cortical areas after CSD induction and rTMS treatment A: Light microscopic appearance (40× magnification) of hippocampal areas; B: The bar graph shows the mean positive TUNEL-labeling neurons in hippocampal regions. The mean number of labeled neurons was significantly increased in CSD and rTMS treated animals in all hippocampal regions in comparison with control animals. Scale bar: 50 μm. Values are presented as mean±SEM. * P<0.05; ** P<0.01; *** P<0.001

**Figure 7.**
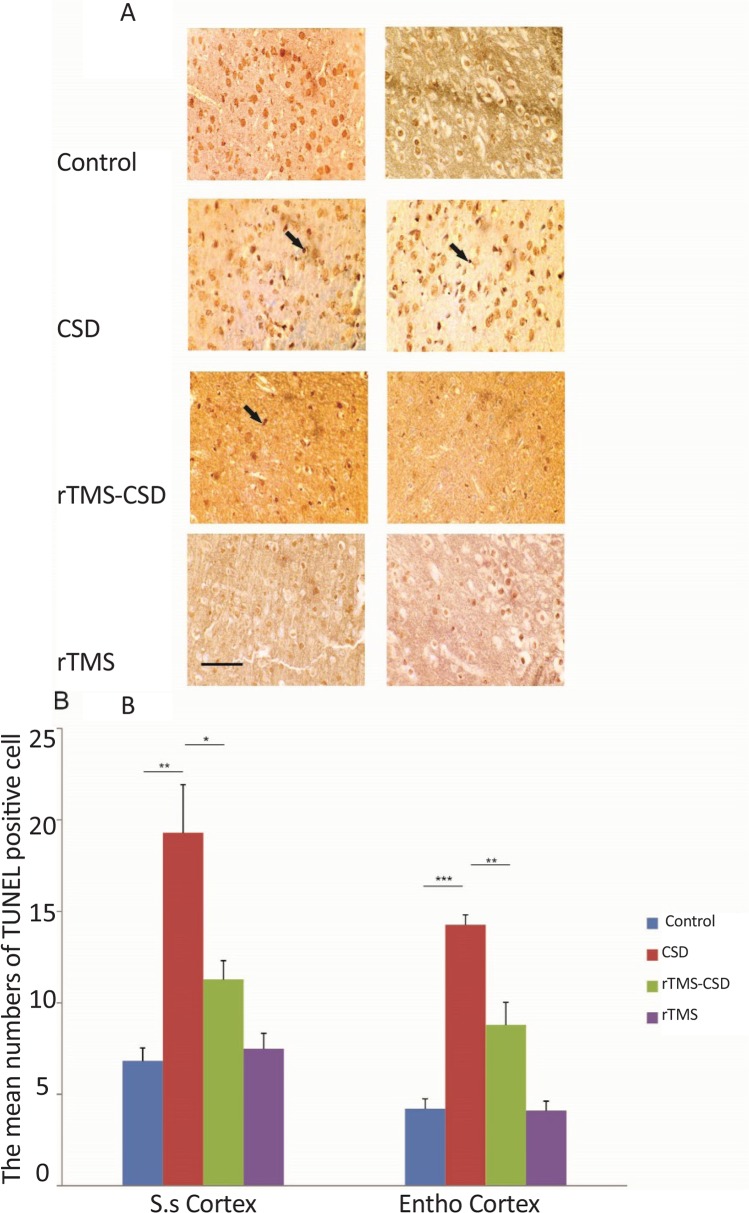
Positive TUNEL-labeling cells indicative of apoptotic cells death in cortical areas after CSD induction and rTMS treatment A: Light microscopic appearance (40× magnification) of cortical areas; B: The bar graph shows the mean positive TUNEL-labeling neurons in cortical regions. The mean number of labeled neurons was significantly increased in the CSD group in all cortical regions compared to the control animals. However, significant reduction in mean number of apoptotic cells was observed in somatosensory and entorhinal cortical region of rTMS treated animals compare to the CSD group. Scale bar: 50 μm .Values are presented as mean±SEM. * P<0.05; ** P<0.01; *** P<0.001

Figure 6A shows TUNEL-positive reacted neuronal cells known as apoptotic cells in various hippocampal regions, including CA1, CA3, and DG. In addition, the mean number of apoptotic neurons in two cortical areas of sumatosensory and entorhinalcortex are presented in Figure 7A. The mean number of TUNEL-reacted cell was significantly increased in the hippocampal CA1 and CA3 areas of both CSD and rTMS treated animals compared to the control animals (P<0.001) (Figure 6B). Histological assessment also revealed a significant increase in the mean number of TUNEL-positive cells in dentate gyrus region of hippocampus in CSD and rTMS treated animals compared to the control animals (P<0.01) (Figure 6B). However, treatment in rTMS-CSD animals significantly decreased TUNEL-reacted neurons in CA1 region compared to CSD animals (P<0.05) (Figure 6B).

Histological assessment in cortical regions revealed a significant increase in the mean number of TUNEL positive cells in somatosensory and entorhinal cortical regions in CSD group compared to the control animals (P<0.01, P<0.001, respectively) (Figure 7B). However, treated animals in rTMS-CSD groups showed significant reduction in the mean number of TUNEL-reacted neurons compared to the CSD animals in somatosensory (P<0.02) (Figure 7B) and entorhinal cortex (P<0.01) (Figure 7B). Table 3 presents the mean number of TUNEL-reacted neurons.

**Table 3.** The mean number of TUNEL-positive cells in different brain regions of experimental groups

**Groups**	**Mean±SEM**				
	
**Regions**	**CA1**	**CA3**	**Dentate Gyrus**	**S.s Cortex**	**Ento Cortex**
Control	2.89±0.32	6.78±1.2	5.49±0.51	6.83±0.69	4.2±0.54
CSD	17.59±1.36	24.34±2.11	12.56±1.34	19.3±2.63	14.2±0.54
rTMS-CSD	17.81±1.8	16.8±0.79	11.83±1.24	11.28±1.03	8.8±1.2
rTMS	5.12±0.35	6.57±1.84	3.2±0.17	7.49±0.84	4.11±0.51

### 3.5. Mean volume-weighted

**Figure 8.**
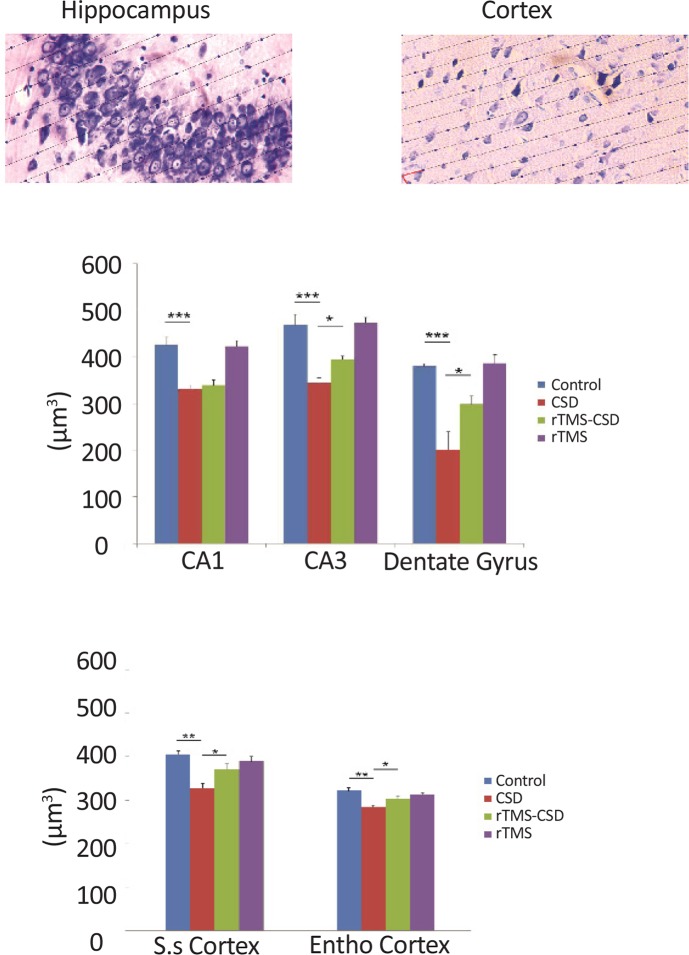
The effects of CSD on the mean volume of normal neurons in hippocampal and cortical areas of rats’ brains A: Light microscopic appearance (40× magnification) of hippocampal and cortical areas during analysis; B: Significant increase in the mean volume of normal neurons in CSD animals in all hippocampal studied areas is observed; C: Mean volume weighted of normal neurons was significantly lower in CSD treated animals in both cortical regions. However, treatment with rTMS showed significantly higher volume weight of normal neurons compared to the CSD animals. Values are presented as Mean±SEM. * P<0.05; ** P<0.01; *** P<0.001

The mean volume of normal neurons was examined in various hippocampal and cortical areas of brain, including CA1, CA3, DG, somatosensory, and entorhinal (Figure 8). A significant reduction in the mean volume of normal neurons in CA1, CA3, and DG hippocampal regions of the CSD group was noticed compared to the control rats (P<0.001) (Figure 8B). In addition, in CA3 and dentate gyrus regions of treated animals, significantly higher volume of normal neuron was observed compared to the CSD animals (P<0.05) (Figure 8B). Histological assessments revealed a significant reduction in the mean volume weight of normal cell in somatosensory as well as entorhinal cortex of the CSD animals compared to the control group (P<0.01) (Figure 8C). However, treatment with rTMS in rTMS-CSD animals in both somatosensory and entorhinal regions resulted in significantly higher volume of normal neurons compared to the CSD group (P<0.05) (Figure 8C).

**Figure 9.**
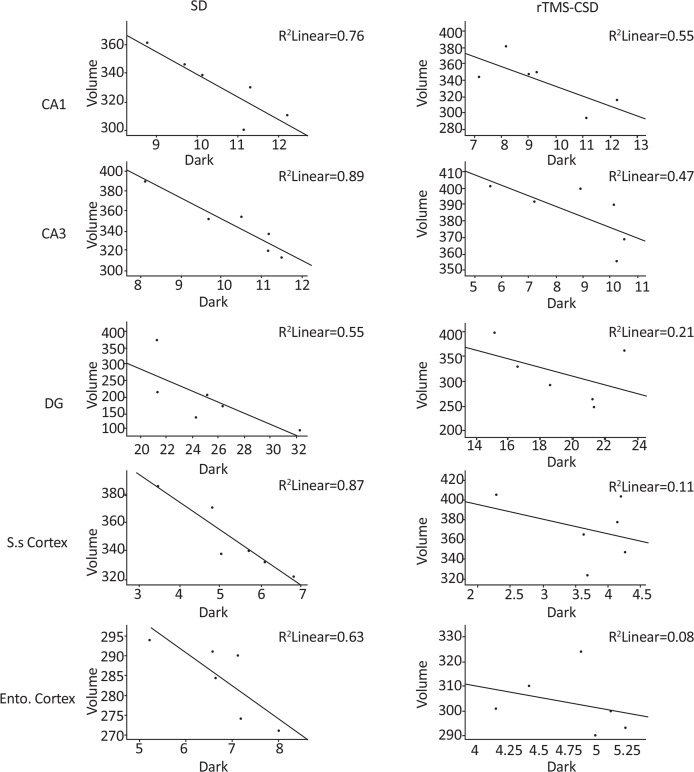
The relationship between the mean normal neurons volume and the mean number of necrotic cells following four consecutive SD and treatment of rTMS in animal brain tissue There was a significant relationship between the mean volume of normal neurons and number of necrotic cells in CA1, CA3, and DG regions of hippocampus. However, no significant correlation between necrotic cells number and volume weight of normal neurons was detected in other brain regions of treated animals of rTMS treated animals compared to SD control animals. Although, significant correlation between the necrotic cells number and volumes weight of normal neurons was observed in CA1 region of hippocampus in treated animals.

The relationship between the number of necrotic cells and volume of normal neurons revealed that the reduction in the volume-weighted mean volume of normal neurons was associated with an increase in the number of necrotic cells in the CA1 (r=0.76, P<0.004) and in CA3 (r=0.89, P<0.05) as well as dentate gyrus (r=0.55, P<0.001) regions following four repetitive SD induction. In addition, same correlation was found in somatosensory (r=0.87, P<0.05), and entorhinal (r=0.63, P<0.05) cortical region of CSD group (Figure 9). However, in treated groups with rTMS, there was no significant correlation between the number of necrotic cells and volume weight of normal neurons. This indicates that a reduction in the volume of normal neurons is not correlated with the rise in the number of dark neurons in CA3, DG, somatosensory, and entorhinal regions. However, significant correlation between changes in the mean volume weight of normal neurons and the number of necrotic cells was revealed in CA1 region of hippocampus (r=0.55, P<0.02) (Figure 9). The mean volume weight of normal neuronal indicated in Table 4.

**Table 4.** The mean volume weight of normal neuronal cell in studied brain regions of experimental groups

**Groups**	**Mean±SEM**				
	
**Regions**	**CA1**	**CA3**	**Dentate Gyrus**	**S.s Cortex**	**Ento Cortex**
Control	425.5±16.8	468.1±22	380.1±5.2	405.6±8.2	322.1±6.5
CSD	331.17±9.3	344.16±11.4	201.4±38.3	328.2±10.1	284.1±3.89
rTMS-CSD	338.9±12.26	394.6±7.4	298.8±17.2	370.5±13.05	303.1±5.07
rTMS	422.3±11.9	472.5±11.94	386.3±18.5	389.3±10.5	313±4.5

## 4. Discussion

We investigated protective properties of rTMS application following CSD induction in rats. Treatment with rTMS could significantly increase the volume of normal neurons in studied brain regions, including CA3, DG, somatosensory as well as entorhinal regions; decreased the mean number of TUNEL-reacted neurons in CA3, somatosensory and entorhinal regions; decreased the mean dark neurons number in DG, somatosensory and entorhinal regions compared to the CSD animals. However, rTMS application showed no significant alternation in memory impairment, induced after CSD induction during four consecutive weeks.

Monkey fMRI studies have established the important role of hippocampus and cortical regions on the memory system (Lee, Lee Wang, & Lin, 1993; Pessoa, Gutierrez, Bandettini, & Ungerleider, 2002; Greicius et al., 2003).

Our histological studies revealed neural death in both cortical and hippocampal regions after four weeks of CSD induction. Repetitive CSD induction increased the mean apoptotic as well as necrotic cells number which leads to the reduction of the mean normal neurons volume in all studied areas of hippocampus as well as in both regions of cortex. Furthermore, the current study indicates a correlation between cell injury/death and cell volume in both hippocampal and cortical regions. The results of the current study confirm previous findings suggesting that repetitive CSD induction could change and or damage neuronal structures (Jafarian et al., 2010; Sadeghian et al., 2012). Neuronal losses in hippocampus could be related to memory deficits in CSD group (Lyeth et al., 1990; Khodaie, Lotfinia, & Lotfinia, 2013). Passive avoidance memory suppression by electrical CSD induction has been reported (Bureš & Burešová 1963). Our memory test indicated that passive avoidance memory will be impaired by the induction of repetitive CSD-like events. This may be due to the neuronal injuries in different regions of brain.

Therefore protection of neuronal cells in different brain region, especially hippocampus could be beneficial for memory improvement. In this regard, rTMS has been widely used for treatment purposes and different studies have shown the neuroprotective effects of long term rTMS application and its usefulness for treatment of neurodegenerative disorders (Post, Müller, Engelmann, & Keck 1999).

TMS has been used for monitoring neuronal activity of cortical region of migraine, along with clinical observations (Aurora, Ahmad, Welch, Bhardhwaj, & Ramadan, 1998). Repetitive TMS (rTMS) application could reduce cortical excitability and enhance phosphene thresholds in migraineurs (Boroojerdi, Prager, Muellbacher, & Cohen, 2000). We observed no significant changes in the case of neuronal injury in rTMS treated animals (rTMS group); these findings agree with the results of previous reports and indicate that rTMS do not cause neuronal injury by itself.

In the current study, reduction in the mean number of injured neurons after treatment may happen due to different mechanisms. Our present work revealed that CSD causes neuronal death in normoxic juvenile brain tissues. Propagation of CSD is correlated with a large influx of ions into the cellular space. Redistribution of ions into the neurons is an energy-dependent process. Consequently CSD initiation is characterized by near-complete breakdown of ion gradients (Dreier, 2011). Mismatch between energy utilization and supply leads to transient energy failure, especially in young rats. This could result in failure of glucose metabolism (Křivánek, 1958) and production of lactic acid (Krivanek, 1961). As rTMS has an electrical nature, it could affect the potential differences of cells and hence change the ion distribution in cellular space. As mentioned above, CSD initiates a large number of ions into cellular space which is concomitant with reduction in glucose supply. If the cell injuries and death is due to the reduction of glucose metabolism, changing potential differences in cell membrane and reducing ions distribution into cellular space by rTMS will decrease cell injuries.

Another possible mechanism for the action of rTMS may be related to the variation of intracellular Ca^2+^ levels. Increase in intracellular Ca^2+^ levels are believed to be involved in neuronal death as well as memory deterioration (Alkon 1984; Aitken, Jing, Young, & Somjen, 1991). Likewise, reduction in Ca^2+^ level inside the cell could improve memory performance. However, rTMS treated animals significantly showed lower degrees of cell injuries, specifically in dentate gyrus and two cortical regions. Thus, rTMS may affect the intracellular Ca^2+^, which is an Ion, and decrease its level which reduces cell injuries. This deduction is along with the above mentioned point that rTMS decreases cell injuries by altering ion distribution.

Our findings support the ability of high frequency rTMS in increasing Cerebral Blood Flow (CBF) in the brain (Paus et al., 1998). Consequently, it has been proposed that rTMS will possibly enhance cortical function by increasing CBF. Faster cellular recovery within the CSD continuum could shift the cell toward apoptosis within the apoptotic-necrotic continuum (Dreier, 2011). In our study, the number of necrotic and apoptotic cells were significantly lower in all cortical and some of hippocampal regions after induction of rTMS, which is in the same line with another report suggested that rTMS may impact neuronal viability (Lisanby & Belmaker 2000). Our findings indicate that rTMS may provide better neuronal protection in cortical regions of treated animals. Hippocampal regions of treated rats showed less significant changes in treated animals than CSD rats.

To summarize, our study indicates that rTMS can be used for the treatment of neurodegenerative disorders. It decreases cell injuries that may be due to different reasons such as changing cortical excitability, reduced ions distributions into cellular space, reduced intracellular Ca^2+^ level, and increased CBF. However, these events are not independent but related to each other. Considering all previous works and our findings, it seems that rTMS reduces ion distribution and hence reduces extra energy consumption in cell spaces. This leads to increased excitability of cortical regions in brain and result in increased CBF. As a result, cell injury and brain damages decrease after rTMS treatment; similar finding have been reported previously in some other neurodegenerative disorders (Khodaie, Saba Lotfinia, & Karimzadeh, 2014).

Stimulation of brain has generated hope in amelioration of neuronal injuries as a novel neuromodulatory treatment through described mechanisms. It is non-invasive, relatively safe and inexpensive. However, the clarification of crucial methodological issues i.e. the optimal stimulating parameters as well as the best site of stimulation need further study before using in clinical trial. Complexity of some neurodegenerative disorders may limit usage of rTMS, which requires more detailed experimental studies. In addition, rTMS could not be administered focally to rodent brain due to limitation in coli size so the whole brain may get stimulated.

Our study confirms previous findings regarding the role of repetitive CSD in cellular death and memory deficits in juvenile rats. We found evidence suggesting protective effects of long term rTMS application in some regions of brain such as cortex and hippocampus in a rat model of CSD. These effects were higher in cortical regions compared to the hippocampus. The present findings implies an attractive and clinically testable hypothesis that rTMS could be beneficial for the improvement of cellular structure in some regions of brain in the case of disorders related to the CSD phenomenon such as migraine. The present findings can be generalized to other kinds of neurodegenerative disorders; however, it needs more investigations and research.
